# Viral and Cellular Factors Involved in Phloem Transport of Plant Viruses

**DOI:** 10.3389/fpls.2013.00154

**Published:** 2013-05-24

**Authors:** Clémence Hipper, Véronique Brault, Véronique Ziegler-Graff, Frédéric Revers

**Affiliations:** ^1^UMR INRA-UDS Virus-Vection GroupColmar, France; ^2^Laboratoire Propre du CNRS (UPR 2357), Virologie Végétale, Institut de Biologie Moléculaire des Plantes, Université de StrasbourgStrasbourg, France; ^3^UMR 1332 de Biologie du Fruit et Pathologie, INRA, Université de BordeauxVillenave d’Ornon, France

**Keywords:** virus, long-distance movement, phloem, viral factors, host factors

## Abstract

Phloem transport of plant viruses is an essential step in the setting-up of a complete infection of a host plant. After an initial replication step in the first cells, viruses spread from cell-to-cell through mesophyll cells, until they reach the vasculature where they rapidly move to distant sites in order to establish the infection of the whole plant. This last step is referred to as systemic transport, or long-distance movement, and involves virus crossings through several cellular barriers: bundle sheath, vascular parenchyma, and companion cells for virus loading into sieve elements (SE). Viruses are then passively transported within the source-to-sink flow of photoassimilates and are unloaded from SE into sink tissues. However, the molecular mechanisms governing virus long-distance movement are far from being understood. While most viruses seem to move systemically as virus particles, some viruses are transported in SE as viral ribonucleoprotein complexes (RNP). The nature of the cellular and viral factors constituting these RNPs is still poorly known. The topic of this review will mainly focus on the host and viral factors that facilitate or restrict virus long-distance movement.

## Introduction

Plant viruses are obligate intracellular parasites living exclusively in the symplast of their hosts. Virus accumulation at high levels throughout the whole plant is a necessary condition for virus survival. Massive titer of virions may facilitate virus transmission from one plant to another, whatever the mode of propagation: by seeds or pollen, by graftings, by mechanical wounds, or by vectors. Viruses are dependent on their hosts to complete their life cycle in the plant, i.e., replication, encapsidation, cell-to-cell movement, and long-distance transport. Therefore, multiple compatible interactions have to be established between viral proteins or virions and cellular factors. The plant reacts to these invaders by developing various strategies to restrict, or even better, eradicate the pathogens. On their side, viruses counteract these defense mechanisms by different ways. The result of this arm race leads to a complete resistance of the plant, if the virus cannot overcome the plant defenses, or to a systemic infection, eventually ending with the host death, if the viral counter defenses are efficient enough to bypass the plant protection system. A wide range of intermediate situations between plant immunity and death can be encountered, which highlights the complexity of interactions that may take place between the virus and the plant.

**Figure 1:**
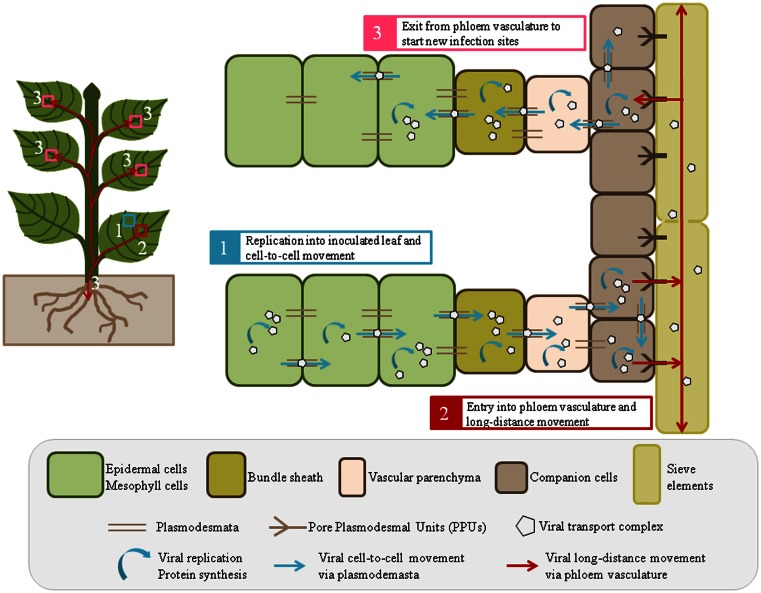
**A general view of virus cell-to-cell and long-distance movement in plant tissues**. After inoculation, mostly into epidermal or mesophyll cells, virions are disassembled for replication and translation of the viral genome (1). Viral proteins, sometimes associated to cellular factors, interact with the viral genome to form the transport complexes (virions or RNP complexes) allowing virus movement from cell-to-cell via plasmodesmata (1). Viral replication and cell-to-cell movement continue in and between nucleate phloem cells, i.e., bundle sheath, vascular parenchyma and companion cells (2). Then, the transport complexes (in the form of virions or RNPs) are loaded into sieve elements for long-distance movement (2), before being finally released into systemic tissues to start a new infection site (3). The whole process requires an effective crossing of successive boundaries between different cell types and leads to systemic infection of the plant.

Virus entry into plant cells, mostly epidermal, and mesophyll, is followed by virion disassembly and genome translation/replication in inoculated tissues. Then, viral transport complexes move from cell-to-cell and on-going replication takes place in the newly infected cells (Figure 1). This short-distance movement requires modification of plasmodesmata (PD) by viral movement proteins (MP; reviewed by Schoelz et al., 2011). Virus transport in phloem tissues encompasses translocation from mesophyll cells to sieve elements (SE) via the successive crossings of the bundle sheath (BS), vascular parenchyma cells (VP), and companion cells (CC). Once in SE, the virus is transported with the phloem sap to distant locations, then it exits from SE to initiate new infection sites and to disseminate efficiently throughout the whole plant (Figure 1). To carry out cell-to-cell and long-distance movements, viruses take advantage of plant existing transport routes, including PD and phloem vasculature, and follow the source-to-sink transportation of carbohydrates (Maule, 1991; Carrington et al., 1996). This review introduces some general features of virus transport in the phloem and addresses the issue of the type of viral complexes that are transported over long-distance. We then focus on viral and host factors shown to play a direct role in virus long-distance movement without affecting multiplication or cell-to-cell movement.

## General Features of Virus Transport in the Phloem

Phloem cells structure and composition reflect their high functional specialization in transporting molecules from source to sink tissues. SE are enucleated cells, modified by selective degradation of organelles, interconnected by wide sieve pores, and forming a low-resistance cellular conduit for elaborated sap flux (Turgeon and Wolf, 2009). SE are maintained alive by an intimate association with CC characterized by a high metabolic activity (Van Bel, 2003). Specialized PD, called Pore Plasmodesmal Units (PPUs), consist of multiple channels on the CC side, and a single channel facing the SE (Oparka and Turgeon, 1999). PPUs exhibit a higher size exclusion limit (SEL) and are therefore more permissive than the PD between mesophyll cells, suggesting that some macromolecules, like proteins or RNAs, may diffuse to SE without specific regulation (Oparka and Turgeon, 1999; Stadler et al., 2005). However, such passive diffusion cannot apply to viral particles (icosahedral or filamentous) or even to infectious ribonucleoprotein (RNP) complexes, formed by the association of viral genome with cellular and/or viral proteins as they are too large to freely move through PPUs.

As shown in Figure 1, viral long-distance movement involves several steps starting from the virus entry into phloem cells (BS, VP, or CC), delivery to SE, transport along SE and exit from SE. This process requires the crossing of successive borders, i.e., mesophyll cell/BS, BS/VP, VP/CC, and CC/SE borders that needs the setting-up of specific interactions between virus and host factors. In the absence of compatible interactions, the virus will be unable to traffic through these gates, making phloem entry, and exit highly restrictive steps for host infection. Several studies on host/virus interactions highlighted that viral transport can be specifically blocked at some of these borders, suggesting precise regulation of the PD permeability at these boundaries (Ueki and Citovsky, 2007 and references therein). The current knowledge on vascular transport infers that virus entry occurs in all vein classes of source leaves, while virus exit is limited to major veins of sink tissues, suggesting different mechanisms for virus loading in and unloading from the phloem (Roberts et al., 1997; Cheng et al., 2000; Oparka and Cruz, 2000; Silva et al., 2002). Virus spread was also shown to follow both internal and external types of phloem, leading to differential directions of transport, either upwards to young sink tissue or downwards to the roots, with the former translocation being faster than the latter (Andrianifahanana et al., 1997; Cheng et al., 2000). Finally, as source leaves preferentially serve sinks with a direct vascular connection (referred to as orthostichy), the viral movement is also predicted accordingly to orthostichy (Roberts et al., 2007). Following symptom appearance and viral accumulation, Roberts et al. (2007) demonstrated the remarkable similarity between *Cauliflower mosaic virus* (CaMV) infection and patterns of photoassimilate distribution in sink organs, indicating that virus movement can be mapped very accurately onto the orthostichy. However, spatial and kinetic analyses of long-distance movement of some viruses revealed that the direction and speed of movement may be different than those of photoassimilates. For instance, *Melon necrotic spot virus* (MNSV) is first transported in melon plants from cotyledons to the roots through the external phloem before being carried to the shoot apex through the internal phloem (Gosalvez-Bernal et al., 2008). The slower rate of virus progression observed in some experimental cases, compared to the speed of photoassimilates, could be explained by additional virus unloading and amplification step in CC before being reloaded into the SE (Moreno et al., 2004; Germundsson and Valkonen, 2006).

From a mechanistic point of view, virus cell-to-cell movement strategies are increasingly well-documented, but far less is known on viral transport mechanisms in vascular system. This lack of knowledge mostly comes from the inaccessibility of this deeply buried tissue, which is difficult to reach, to handle, and to study. In addition, collecting phloem sap to identify virus phloem partners may be challenging or even infeasible depending on the host. At last, as the efficiency of cell-to-cell movement influences the long-distance transport of virus, these two interconnected processes are sometimes difficult to distinguish. Consequently, the identification of viral and host factors specifically required for virus long-distance transport is sometimes misinterpreted and still represents a challenge. Nevertheless, during the last 10 years, a growing body of data has shed light on factors involved in virus vascular transport, in particular the viral determinants promoting the long-distance spread and some host factors facilitating or restricting this process.

## Viral Complexes Transported Over Long-Distance

The nature of the viral complexes transported in sieve tubes from inoculated to non-inoculated leaves is an important question to address to better understand the mechanisms by which viruses invade whole plants. Two viral forms of transport have been described: virions, protecting the genome by a shell formed by capsid protein (CP) subunits assembly, and RNP complexes, in which the viral genome is associated with viral and/or cellular proteins. As described in more details in the following section, the requirement of a functional CP for systemic movement is common but not universal. Although this occurrence is usually associated with the need to produce virions, the CP can also be required to form RNP complexes. The nature of the complexes involved in long-distance transport of different viral species is described thereafter, emphasizing the central role of the CP (see also Table A1 in Appendix).

Viral particles have been reported to be the exclusive long-distance moving form of different virus species belonging to distinct genera like *Potexvirus, Alfamovirus*, *Cucumovirus*, *Closterovirus*, *Mastrevirus*, *Begomovirus*, *Dianthovirus*, *Carmovirus*, *Necrovirus, Tobamovirus*, *Sobemovirus*, and *Benyvirus* (Table A1 in Appendix). For other virus species, although the absolute requirement of a functional CP for virus long-distance transport has been demonstrated, it is still unknown whether virions, or CP-associated RNP complexes, are involved in this process. This concerns members in the *Potexvirus*, *Cucumovirus, Bromovirus*, *Tospovirus, Closterovirus*, *Curtovirus, Polerovirus*, and *Potyvirus* genera (Table A1 in Appendix).

Interestingly, *Potato mop-top virus* (PMTV, *Pomovirus*) was shown to move simultaneously in the form of RNP complexes and virions, the three RNAs of this multipartite virus being transported in different forms (see also below in the next section; McGeachy and Barker, 2000; Savenkov, 2003; Torrance et al., 2009, 2011; Wright et al., 2010). *Brome mosaic virus* (BMV) is another example for which systemic movement of each of the three genomic RNA may occur in different forms, and may involve constitution of RNP complexes with cellular factors. Gopinath and Kao (2007) showed that BMV-RNA-3 was able to move over long-distance without the assistance of any viral protein whereas BMV-RNA1 and RNA2 were also competent for systemic movement but needed the MP. Whether transport of BMV-RNAs is only required for the initial step of virus infection or is thereafter an alternative mode of virus transport together with virions, previously shown to be required for systemic spread, requires further investigations (Sacher and Ahlquist, 1989; Flasinski et al., 1997). For other viruses, the viral form that traffics in the vasculature may depend on the host plant and the degree of virus-host adaptation. *Bean golden mosaic virus* (BGMV, *Begomovirus*) for example, moves in *N. benthamiana* and in its natural host, *P*. *vulgaris*, in a CP-dependent manner, most probably virions, but this virus can also be transported in beans, although less efficiently, as CP-independent RNP complexes (Jeffrey et al., 1996; Pooma et al., 1996). A similar bi-modal process was observed in *N. benthamiana* for *Tomato golden mosaic virus* (TGMV), another *Begomovirus* (Pooma et al., 1996), and in *Nicotiana* species for the NM isolate of *Tobacco rattle virus* (TRV, *Tobravirus*) lacking the CP encoded by RNA2 (Swanson et al., 2002; Macfarlane, 2010).

The CP requirement for virus long-distance spread is certainly more a general rule than an exception. Some viruses, however, do not need the CP to move systemically. Mutations introduced in the viral genome of *Tomato leaf curl virus* (ToLCV, *Begomovirus*) (Padidam et al., 1995, 1996) and *Tomato bushy stunt virus* (TBSV, *Tombusvirus*) (Scholthof et al., 1993; Desvoyes and Scholthof, 2002; Qu and Morris, 2002) inhibit synthesis of the CP and formation of virions, but do not alter virus transport in non-inoculated leaves. The presence of the CP, however, is accelerating virus transport resulting in more severe symptoms on the infected plants (Desvoyes and Scholthof, 2002; Qu and Morris, 2002; Manabayeva et al., 2013). However, long-distance movement of TBSV in *N. benthamiana* occurs independently of CP upon root inoculation (Manabayeva et al., 2013). A very singular case is represented by umbraviruses (*Groundnut rosette virus*, GRV, and *Pea enation mosaic virus-2*, PEMV-2) that lack a CP-encoding gene and move naturally in the form of RNP complexes. These complexes are formed by the association of the viral RNA genome with the viral protein encoded by ORF3 and the host nuclear fibrillarin (see below; Ryabov et al., 2001; Taliansky et al., 2003; Kim et al., 2007a,b; Canetta et al., 2008).

Viral encapsidation was considered for many years as a means to protect the RNA genome from the potential harshness of the phloem environment. Actually, no RNAse activity has ever been found in this plant compartment (Sasaki et al., 1998; Doering-Saad et al., 2002), but an entire machinery for a functional 26S proteasome was identified in pumpkin sap exudates (Lin et al., 2009). Moreover, recent proteome studies identified aminopeptidases and proteases in sieve tube sap of pumpkin and *A. thaliana* (Lin et al., 2009; Batailler et al., 2012) suggesting that viruses may need to develop strategies, like the recruitment of cellular factors, to protect their virions or RNP complexes from these proteolytic enzymes.

## Viral Determinants Which Facilitate Long-Distance Movement

The CP is obviously the major viral determinant involved in virus long-distance movement but several other viral proteins were also shown to play a role in this process (reviewed in Waigmann et al., 2004; Ueki and Citovsky, 2007). Here, we mainly emphasize recent data on these proteins and highlight the importance of RNA silencing suppressors (RSS) for efficient systemic spread in the plant.

### Capsid protein

As described in the previous section, CP requirement is often linked to the necessity to form viral particles for systemic transport. However, CP domains distinct from those required for viral encapsidation were reported to participate to virus long-distance movement.

This is the case for potyviruses for which the N- and C-terminal CP domains are dispensable for virus genome encapsidation, but essential for virus long-distance movement (Dolja et al., 1994, 1995). In addition to being a resistance breaking determinant, the N-terminal domain of the CP (CP-N) was shown to be a host- and strain-specific long-distance movement determinant for *Potyviridae* family members (Salvador et al., 2008; Decroocq et al., 2009; Tatineni et al., 2011a). Similarly, the C-terminal domain of the CP of two *Tombusviridae* family members, *Olive latent virus-1* (OLV-1, Pantaleo et al., 2006), and *Carnation ringspot virus* (CRSV, Sit et al., 2001), was reported to be specifically involved in systemic movement but not in particle formation, even though virions are necessary for vascular transport of these two viruses (Table A1 in Appendix). The most likely hypothesis regarding the role of these CP domains in virus long-distance transport is their exposure on the external surface of the virion allowing them to directly interact with host factors. A recent study showing that a CP domain essential for the systemic movement of the *Cucumber mosaic virus* (CMV, *Cucumovirus*) forms a loop on the surface of the virion reinforces this hypothesis (Salánki et al., 2011).

Regarding the role of virus assembly in long-distance movement, it has been shown for two viruses belonging to the *Tombusviridae* family, the Carmovirus *Turnip crinkle virus* (TCV, Cao et al., 2010), and TBSV (Qu and Morris, 2002), that particles are dispensable for loading into vascular tissues, but are essential for efficient vascular egress. Different molecular mechanisms may therefore control the entry and the exit of viral genomes into and from the SE. These data are in agreement with the fact that some host factors (see below) specifically control viral phloem exit.

### Movement protein

Plasmodesmata are small channels allowing the movement of molecules between plant cells by forming a cytoplasmic continuum known as symplasm (reviewed in Lucas et al., 2009; Maule et al., 2011). These specialized channels used by viruses to move from cell-to-cell, are however too small to allow passive transport of viruses. MPs are therefore synthesized by viruses to increase PD permeability (reviewed by Scholthof, 2005; Benitez-Alfonso et al., 2010; Niehl and Heinlein, 2010; Schoelz et al., 2011). Interestingly, some viruses like the monopartite and bipartite geminiviruses, CMV, and the *Red clover necrotic mosaic virus* (RCNMV, *Dianthovirus*) require MPs for long-distance movement (reviewed in Waigmann et al., 2004; Ueki and Citovsky, 2007). Other studies showed that distinct domains of the MPs are involved in virus cell-to-cell or in long-distance transport. For instance, the C-terminus of the non-structural protein (NSm) of the *Tomato spotted wilt virus* (TSWV, *Tospovirus*), which is the MP of TSWV, is essential for systemic movement (Lewandowski and Adkins, 2005; Li et al., 2009). A similar situation is described for BMV for which the C-terminal domain of the MP is not essential for virus cell-to-cell movement but required for long-distance transport (Takeda et al., 2004).

### Triple gene block proteins

The triple gene block (TGB) proteins, encoded by three partially overlapping ORFs in nine genera within the *Alphaflexiviridae*, *Betaflexiviridae*, and *Virgaviridae* families, and in the unassigned genus *Benyvirus*, are essential for virus cell-to-cell movement (reviewed in Morozov and Solovyev, 2003; Verchot-Lubicz et al., 2010; Solovyev et al., 2012). Some viruses require the TGB1 protein for systemic spread (hordei-like viruses, *Virgaviridae*) while other viruses (potex-like viruses, *Alphaflexiviridae*) are dependent on the CP in addition to the TGB1 protein, for both cell-to-cell and long-distance movement (reviewed in Verchot-Lubicz et al., 2010).

TGB1 is a multifunctional protein that has, among others, the property to bind single stranded RNA (ssRNA) and form RNP complexes (Lough et al., 2000). This characteristic was further studied for hordeiviruses for which RNP complexes proved to be competent for short- and long-distance viral transport (Lim et al., 2008). The hordeiviral TGB1 proteins differ from the potexvirus-like TGB1 in having a longer N-terminal extension with positively charged amino acids. This extension consists of two structurally and functionally distinct domains, referred to as the N-terminal (NTD) and the internal (ID) domains. TGB1-NTD is dispensable for movement between cells, but is essential for vascular transport (Makarov et al., 2009). The structurally disordered NTD and the structured ID domains are both interacting with ssRNA and could play the role of an RNA chaperone stabilizing RNP complexes in the phloem, thereby functioning like the CP in potex-like viruses (Makarov et al., 2009).

Recently, TGB1-NTD of *Poa semilatent hordeivirus* (PSLV) was shown to contain targeting sequences for the nucleolus and cajal bodies (CB) (Semashko et al., 2012a). PSLV TGB1 interacts *in vitro* and *in vivo* with fibrillarin and coilin (Semashko et al., 2012a,b), two proteins localized respectively in the nucleolus and in CB. As the nucleolar fibrillarin is known to play an essential role in long-distance movement of an *Umbravirus* (see below), interaction with fibrillarin may represent a more general mode of action promoting viral systemic trafficking. Similar *in vitro* interactions between PMTV TGB1 and fibrillarin were reported (Wright et al., 2010; Torrance et al., 2011) but relevance of these interactions in long-distance movement of hordeiviruses remains to be assessed (Solovyev et al., 2012).

A very unusual and complex situation was described for PMTV (*Pomovirus* genus, *Virgaviridae* family*)* regarding long-distance transport of its three genomic RNAs. All of them require the TGB1 to move systemically. While two of the genomic RNAs can spread in the absence of the CP, the third RNA encoding the CP needs the minor capsid protein (CP-RT) for phloem transport (Torrance et al., 2009). Deletion of the N-terminal domain of PMTV TGB1 did not affect the capacity to self-interact, to interact with CP-RT nor with the viral RNA (Wright et al., 2010). This domain could therefore be required for the binding to a host factor involved in viral systemic transport (Wright et al., 2010; Torrance et al., 2011). Together with additional observations, these data suggest that the CP-encoding RNA moves over long-distance packaged into virions decorated with both TGB1 and the CP-RT protein at one extremity (Torrance et al., 2009, 2011). This example illustrates perfectly the situation where a virus can reach non-inoculated leaves using different viral forms (virus particles and CP-independent RNP complexes) (see also “Viral complexes transported over long-distance”).

### Potyvirus VPg

Besides its role in virus replication (Jiang and Laliberté, 2011), the viral genome-linked protein (VPg) of potyviruses is also involved in virus movement. Several studies showed that VPg is the breaking determinant of the resistance based on virus long-distance movement restriction. This function of the VPg was demonstrated for *Tobacco etch virus* (TEV) in tobacco (Schaad et al., 1997) and in different plant species for *Potato virus A* (PVA), *Nicandra physaloides* (Rajamäki and Valkonen, 1999), a diploid potato hybrid (Hämäläinen et al., 2000), and *Solanum commersonii* (Rajamäki and Valkonen, 2002). For PVA, one amino acid change in the central domain of the VPg is sufficient to restore viral long-distance movement, although this resistance bypass is host-specific (Rajamäki and Valkonen, 1999, 2002). Using grafting experiments, it was also shown that the PVA long-distance movement restriction was likely due to the absence of virus loading into SE (Hämäläinen et al., 2000; Rajamäki and Valkonen, 2002). The VPg is covalently linked to the 5’ end of the viral RNA and is exposed at one extremity of the virion. It is therefore accessible for interaction with proteins and in particular with phloem host factors involved in virus movement (Puustinen et al., 2002). Consequently, any mutation in either the VPg or the host factors disrupting these interactions may abolish virus long-distance movement, thereby conferring resistance to the host. This is exemplified by a mutation in the N-terminal part of *Turnip mosaic virus* (TuMV) VPg that abolishes its interaction with the cellular protein PVIP (see “host factors involved in phloem transport of potyviruses”) and results in a strong delay in systemic infection (Dunoyer et al., 2004).

### Potyvirus 6K2

The small 6K2 protein of potyviruses is an integral membrane protein associated to VPg in endoplasmic reticulum-derived membranes (Schaad et al., 1997; Léonard et al., 2004) forming cytoplasmic vesicles which are viral replication sites (Cotton et al., 2009). Rajamäki and Valkonen (1999) showed that, in addition to the VPg (see above), the 6K2 of PVA is a virulence determinant in *N. physaloides* enabling the virus to overcome the resistance that restricts PVA long-distance movement in this host. One amino acid change in the N-terminal sequence of 6K2 (6K2-N) was indeed sufficient to restore virus systemic spread. As 6K2-N is located on the cytoplasmic side of the membrane (Schaad et al., 1997b), it can potentially interact with viral or host factors implicated in potyvirus long-distance movement. In particular a coordinated role for the VPg and the 6K2 proteins in PVA vascular transport can be envisaged. Whether the 6K2 protein from other potyviruses participates to virus long-distance movement needs to be addressed.

### Umbraviruses ORF3

Another well characterized viral protein involved in virus long-distance movement is the *Umbravirus* ORF3 protein. Umbraviruses, which do not encode a CP are unable to produce typical virus particles (Taliansky and Robinson, 2003). Instead, they move as filamentous RNP complexes formed by the interaction between ORF3 protein and viral RNA (Taliansky et al., 2003). ORF3 protein of GRV is able to translocate heterologous viral RNA through the whole plant (Ryabov et al., 1999, 2001). In all cell types, and particularly in phloem cells, ORF3 protein accumulates in cytoplasmic inclusions containing filamentous RNP particles (Taliansky et al., 2003). A remarkable shuffling of ORF3 protein from the cytoplasm to the nucleus is essential for virus movement (Ryabov et al., 1998, 2004). Indeed, the ORF3 protein traffics to the nucleolus via a mechanism involving the reorganization of CBs into multiple CB-like structures (CBL) and their fusion with the nucleolus (Kim et al., 2007a,b). In these nuclear structures, the interaction between the ORF3 protein and the nuclear protein fibrillarin mediates the relocalization of fibrillarin to the cytoplasm where it is integrated into viral RNP complexes together with the ORF3 protein (Kim et al., 2007a,b). A direct interaction between the leucine-rich domain of the ORF3 protein and the Glycine- and Arginine-Rich domain of fibrillarin was further demonstrated (Kim et al., 2007a). Functional analysis using ORF3 protein mutants and *N. benthamiana* silenced for fibrillarin expression, revealed a correlation between the ORF3/fibrillarin interaction, the formation of RNP complexes, and the virus long-distance transport (Kim et al., 2007a,b). Finally, *in vitro* reconstituted ORF3 protein/fibrillarin/viral RNA complexes were shown to be infectious *in planta* suggesting that no additional viral or plant factor is required for *Umbravirus* long-distance movement (Canetta et al., 2008).

### Long-distance viral determinants of phloem-limited viruses

#### Closterovirus proteins

Closteroviruses form long filamentous particles bearing a tail composed of several proteins involved in cell-to-cell transport (Napuli et al., 2003; Peremyslov et al., 2004). The tail of the particle was therefore proposed to be a specialized transport device and not merely a protection for viral RNA. The MP Hsp70h of *Beet yellows virus* (BYV) is one of the tail components that targets the cell periphery and PD (Prokhnevsky et al., 2005). Hsp70 interacts with p20, a protein which was shown, by atomic force microscopy on BYV particles, to be also located at the tip of the tail (Prokhnevsky et al., 2002; Peremyslov et al., 2004). This interaction may therefore provide a PD docking site for p20. P20 has a moderate effect on virus local spread, whereas it is essential for virus long-distance movement (Prokhnevsky et al., 2002). By it localization, p20 may facilitate entry into or exit from the phloem via direct or indirect modifications of the PPUs connecting CC and SE (Prokhnevsky et al., 2002). P20 could also function to stabilize virions inside the phloem sap or could eventually inactivate phloem antiviral plant defense response (Prokhnevsky et al., 2002; Peremyslov et al., 2004).

Another BYV protein involved in virus long-distance movement is the leader proteinase (L-Pro) which functions in RNA replication and in polyprotein processing (Peng et al., 2003). Both non-conserved N-terminal and conserved C-terminal domains of L-Pro seem to be involved in BYV long-distance movement. However, in contrast to the p20 protein, L-Pro is not associated to virions and its mode of action in virus long-distance transport remains to be determined.

In the case of *Citrus tristeza virus* (CTV), three non-conserved genes corresponding to the p33, p18, and p13 proteins can be deleted without affecting the ability of the virus to systemically infect the more susceptible citrus trees (Tatineni et al., 2008). In some others citrus species, one or two of these genes are essential for systemic infection (Tatineni et al., 2011b). Two additional genes encoding p20 and p6 proteins are suspected to be required for virus wide spread throughout citrus trees (Tatineni et al., 2008). However, the BYV p6 homolog was considered by Alzhanova et al. (2000) as a MP.

#### Polerovirus P4 and readthrough proteins

Polerovirus virions are composed of the major coat protein of 23 kDa and a minor component, the readthrough protein (RT). This protein of about 74 kDa is a C-terminally extended form of the CP produced by occasional suppression of the CP termination codon. It gets processed by an unknown mechanism into a 54 kDa protein (RT*), which is found incorporated into virions. CP, RT, and RT* are involved in virus long-distance transport (Bruyère et al., 1997; Brault et al., 2000; Peter et al., 2008; Brault and Boissinot, personal communication). Particles were detected in PD connecting nucleated phloem cells and SE suggesting that virions are the phloem mobile device of poleroviruses (Esau and Hoefert, 1972; Shepardson et al., 1980; Mutterer et al., 1999). Moreover, virions were observed in sap collected from cucumbers infected with the polerovirus *Cucurbit aphid-borne yellows virus* (CABYV, Brault and Boissinot, personal communication). Mutations in the CP gene that disrupt virion formation inhibit systemic transport (Brault et al., 2003), reinforcing the role of virus particles in polerovirus long-distance movement. Mutations or deletions affecting synthesis and/or incorporation of the RT* protein into *Potato leafroll virus* (PLRV) virions reduce or completely inhibit virus systemic movement, depending on the hosts (Peter et al., 2008). Furthermore, the C-terminal part of the RT protein was reported to be important to confine PLRV to the phloem tissue (Peter et al., 2009).

P4, on the other hand, is a non-structural protein sharing biochemical and cellular characteristics of conventional cell-to-cell MPs like its ability to bind ssRNA, target PD, increase PD SEL, form homodimers and be phosphorylated (Tacke et al., 1993; Schmitz et al., 1997; Sokolova et al., 1997; Hofius et al., 2001; De Cilia and Ziegler-Graff, personnal communication). P4-defective polerovirus mutants are still able to replicate in protoplasts (Ziegler-Graff et al., 1996), but are impaired in their ability to move over long-distances although only in some hosts. As the involvement of P4 in cell-to-cell movement has not been precisely addressed yet, essentially by the lack of experimental system, it is possible that the impaired vascular movement of P4 mutants originates from a delay in cell-to-cell transport (Lee et al., 2002; Ziegler-Graff and Brault, unpublished results). A working hypothesis could be the co-existence of two movement pathways, one dependent and the other independent of P4 (Ziegler-Graff et al., 1996). Additional experiments are required to decipher the precise role of P4 in polerovirus movement.

### RNA silencing suppressors

The discovery of RNA silencing and the concomitant characterization of the RSS led to shed new light on long-distance trafficking of viruses in the phloem. In higher plants and insects, RNA silencing is an adaptive major defense mechanism against viruses based on the production of virus-specific short interfering RNA (siRNA) able to target cognate RNA sequences. These siRNA are generated from double-stranded RNA (dsRNA) by Dicer-like enzymes (DCL) and then recruited by RNA-induced silencing complexes (RISC) containing an ARGONAUTE (AGO) effector protein. siRNA guide the sequence-specific cleavage by AGO1 of homologous targets (Ding and Voinnet, 2007). Interestingly, RNA silencing is a non-cell autonomous process known to function through a silencing signal able to spread through PD from the initial cell, where silencing was triggered, to the adjacent cells, but also over long-distance following the plant vasculature (Kalantidis et al., 2008). The silencing signal travels ahead of the viral infection front, immunizing the recipient tissues, and preventing the systemic spread of the virus toward upper non-infected leaves (Schwach et al., 2005; Ding and Voinnet, 2007). The silencing signal is amplified by host RNA-dependant RNA polymerases (RDR; Schwach et al., 2005) thereby generating new sources of dsRNA that are processed into secondary siRNA (Wang et al., 2010). The identity of the mobile silencing signal was recently confirmed as being a small RNA duplex (Dunoyer et al., 2010). Thus, the siRNA signal does not only reduce viral accumulation in the initially infected cell, but can also move ahead of the virus, restricting subsequent virus cell-to-cell movement and systemic trafficking.

To counter this host defense, viruses have developed diverse strategies by encoding RSS that interfere with the activity of various compounds of the silencing pathway (Burgyán and Havelda, 2011). Many RSS were previously known as virulence factors able to intensify symptoms or promote systemic infection (Díaz-Pendón and Ding, 2008). But RSS are often multifunctional proteins that display essential roles in the infection process like replication, coating, movement, and pathogenesis, which may hinder their study. Since the discovery of RSS almost 15 years ago, two main strategies of inhibition of the silencing pathway have emerged. The first one involves binding to the small RNA duplex, thus preventing siRNA loading into the RISC complex (Lakatos et al., 2006; Mérai et al., 2006). This process also inhibits the spread of the silencing signal to neighboring cells (Silhavy et al., 2002) and to distant parts of the plant (Dunoyer et al., 2010). The second mode of action of RSS targets the effector protein AGO1 that functions cell-autonomously (Dunoyer et al., 2010). The mechanism inhibits both the primary and the secondary siRNA-guided cleavage, impairing the generation of new antiviral silencing signals. This section will focus on RSS that were reported to promote viral long-distance movement and will attempt to correlate the mode of action of the RSS with their requirement for viral spread.

#### Tombusvirus P19

The P19 protein encoded by TBSV (*Tombusviridae*) is essential for long-distance spread in spinach and pepper plants, while it is dispensable for systemic infection of *N. benthamiana* and *N. clevelandii*, suggesting that P19 displays an essential host-dependent role in systemic movement (Scholthof et al., 1995). However, a recent study showed that P19 is required for systemic infection in *N. benthamiana* upon root inoculation with TBSV, inferring that silencing in the inoculated root cells is more immediate and effective than in leaves (Manabayeva et al., 2013). During the following years, several studies have characterized P19 as an RSS. First, expression of P19 was able to prevent RNA silencing in the upper leaves of an infected plant, but P19 could not reverse established RNA silencing, suggesting that P19 compromised the systemic spread of a signal needed for activation of RNA silencing (Voinnet et al., 1999). Molecular studies demonstrated that P19 binds dsRNA of 21 bp with a high affinity (Silhavy et al., 2002). Crystallographic data further confirmed that P19 dimers can specifically sequester siRNA duplexes (Vargason et al., 2003; Ye et al., 2003). Recent findings also showed that P19 interferes with the spread of siRNA duplexes, which were identified as the signal of systemic RNA silencing (Dunoyer et al., 2010). Finally, elegant *in situ* hybridization experiments revealed that the P19 of *Cymbidium ringspot virus* (CymRSV, *Tombusvirus*) promoted virus exit from vascular tissues into the surrounding cells and the subsequent systemic infection of the upper leaves (Havelda et al., 2003).

#### Cucumovirus 2b

The second best studied RSS is the 2b protein encoded by CMV. This small protein of 100 amino acids encoded by a cryptic ORF was found to enhance virus systemic spread in a host-dependant manner. The Q-strain of CMV mutant lacking the 2b ORF (CMV-Δ2b) was unable to systemically infect cucumber plants although it accumulated in inoculated cotyledons (Ding et al., 1995). In tobacco plants however, the same CMV-Δ2b virus was able to spread systemically to upper leaves. A similar 2b-deletion mutant of the severe Fny-strain of CMV remained infectious in tobacco and *N. benthamiana*, but its movement dynamics was affected in both inoculated and systemic leaves. Moreover, infected plants did not develop symptoms (Soards et al., 2002; Ziebell et al., 2007). These experiments argue for an effect of both the virus strain and the host in CMV long-distance movement and symptom induction.

Additional studies showed that the 2b protein was able to prevent the spread of the systemic silencing signal (Brigneti et al., 1998; Guo and Ding, 2002), facilitating infection of distal parts of the plant. Information unraveling the mode of action of the 2b protein came from genetic studies on *A. thaliana* wild-type and *rdr* mutants infected with a 2b-deficient CMV mutant (Diaz-Pendon et al., 2007). These studies showed that the 2b protein expressed from the CMV genome severely reduced the accumulation of viral secondary siRNA produced by RDR1 or RDR6 (Wang et al., 2011). In addition, several functional studies also revealed that the 2b protein displays a dual mode of silencing inhibition. First, by physically interacting with AGO1, the 2b protein is able to block the slicing activity of AGO1 (Zhang et al., 2006). Secondly, by binding directly to siRNAs duplexes it could prevent the antiviral activity of the small RNA (Goto et al., 2007). Although the specific contribution of each function of the 2b protein during the CMV infection process is hard to assess presently, it is clear that the CMV 2b protein facilitates short- and long-distance spread of the virus *in planta*.

#### Potyvirus HC-Pro

Fundamental studies on potyviruses based on mutagenesis showed that the central part of TEV HC-Pro, but not the N- and C-terminal domains, is required for viral long-distance movement and replication-maintenance functions (Dolja et al., 1993; Cronin et al., 1995; Kasschau and Carrington, 2001). Further experiments correlated both replication and long-distance trafficking functions with the silencing suppression activity of HC-Pro (Kasschau and Carrington, 2001). Conversely to TEV HC-Pro, the N-terminal domain of the HC-Pro of *Tobacco vein mottling virus* (TVMV) and *Papaya ringspot virus* (PRSV) was essential to inhibit RNA silencing (Yap et al., 2009).

Long-distance movement deficiency of *Plum pox potyvirus* (PPV) in tobacco plants could be complemented in transgenic plants expressing the 5′ terminal region of the TEV genome (containing the HC-Pro coding sequence), but not in plants transformed with a mutated form of TEV HC-Pro (Sáenz et al., 2002).

Interestingly, a TuMV mutant, deficient in HC-Pro and unable to move systemically in *A. thaliana* wild-type plants, regained long-distance movement when both RDR1 and RDR6 were knocked out (Garcia-Ruiz et al., 2010). This strongly supports the hypothesis that HC-Pro promotes systemic infection by suppressing an siRNA-dependent activity.

Functional studies on the RSS activity of HC-Pro showed that the protein is able to bind siRNA duplexes and thereby impairs loading of new siRNA into RISC complexes and further compromises the amplification step by the plant RDRs (Lakatos et al., 2006). This fundamental siRNA loading into RISC can also be inhibited indirectly as HC-Pro has the potential to suppress the 3′-terminal methylation of siRNA mediated by HEN-1 (Ebhardt et al., 2005; Jamous et al., 2011). Cleavage activity of programed RISC was however not affected.

Recently, a transcription factor RAV2 induced by the ethylene defense pathway was identified as being required for suppression of silencing mediated by HC-Pro (Endres et al., 2010).

#### Carmovirus P38 (TCV)

Turnip crinkle virus CP (also referred to as P38) is a multifunctional protein involved in virus assembly, but also in suppression of RNA silencing and in induction of R gene-mediated resistance (Cohen et al., 2000; Qu et al., 2003; Choi et al., 2004). Its direct role in long-distance movement was investigated by uncoupling packaging and RSS functions using a genetic approach and a GFP-labeled TCV deleted of its CP gene (Deleris et al., 2006). The deficient encapsidation function was provided by transgenic plants expressing a TCV CP mutant unable to suppress RNA silencing. The successful sap inoculation of P38-expressing plant by this trans-encapsidated GFP-TCV-ΔCP mutant showed for the first time, that TCV CP promotes systemic trafficking by its RNA silencing suppression activity in an assembly independent way. Similarly, the N-terminal domain of the CP eliciting R gene-mediated resistance is not involved in RNA silencing suppression (Choi et al., 2004). More recently, Cao et al. (2010) reinvestigated the genetic requirements for TCV long-distance movement using *A. thaliana* mutants lacking antiviral silencing activity (*dcl2dcl3dcl4*). By monitoring the propagation of several TCV CP mutants in such plants they observed that only mutants bearing a functional silencing suppression activity could invade the vasculature of systemic leaves. Moreover, in this genetic background, all viral mutants unable to form particles remained restricted to the vascular tissues of upper leaves. These observations suggested the existence of two barriers that could block the systemic spread of TCV. The first barrier would be at the entry point into the vascular bundles and could be overcome by the CP, even if the protein is deficient for encapsidation. The second barrier corresponding to the exit from the vascular bundles of systemically infected leaves would be dependent on virus assembly. The apparent discrepancy between these data and those presented by Deleris et al. (2006) could arise from different experimental conditions (inoculum, organ analyzed) (Cao et al., 2010).

Regarding the mechanism of action of CP as RSS, several studies pointed out different properties that would highlight a possible dual function, reminding the case of the CMV 2b protein (see above). Mérai et al. (2006) showed that TCV CP is able to inhibit the processing of dsRNA into siRNA and that it binds dsRNA in a size-independent manner. This infers that CP inhibits the generation of siRNA from hairpin transcripts by competing with DCL for long dsRNA. This hypothesis is in agreement with the genetic evidence showing that DCL4, which confers the primary antiviral activity in *A. thaliana*, is inhibited in TCV-infected cells (Deleris et al., 2006). Moreover, a recent study demonstrated that the TCV CP is able to interact with AGO1 by mimicking the cellular GW/WG repetitive motif of AGO1-interacting proteins, and thereby interfering with AGO1 functions (Azevedo et al., 2010).

#### Closterovirus

The genome organization of *Closteroviridae* displays complex and diversified coding capacities. Among the 10 proteins encoded by BYV, two were reported to be enhancers of replication and involved in long-distance movement, the L-Pro and the p21 proteins. Only the latter exhibited silencing suppression activity (Reed et al., 2003). Biochemical studies showed that p21 binds siRNA duplexes (Chapman et al., 2004). The crystal structure of p21 revealed an octameric ring architecture with a large central cavity likely involved in RNA-binding (Ye and Patel, 2005). Although the structure bears no similarity with that of the TBSV p19 RSS, their activity might be very similar by sequestering siRNA duplexes.

The situation is very different for the phloem-restricted CTV. Three silencing suppressors were identified among the 12 proteins encoded by CTV: p20, a homolog of BYV p21, CP, and p23 (Lu et al., 2004). P23 which is unique among closteroviruses (Dolja et al., 2006) is an RNA-binding protein with a Zn-finger motif (López et al., 2000). P23 and p20 inhibit intercellular silencing while p20 and the CP act intracellularly on RNA silencing. Among these proteins, only p20 is potentially involved in long-distance spread in citrus, but no molecular data are yet available to explain its mode of action.

#### Beet necrotic yellow vein virus p14

By a point mutagenesis approach, the reported RSS of *Beet necrotic yellow vein virus* (BNYVV, *Benyvirus*), p14, was shown to be essential for virus long-distance movement in *Beta macrocarpa* (Chiba et al., 2013) while it was dispensable for replication or virus cell-to-cell trafficking. P14 is a zinc-finger cysteine-rich protein that targets the nucleolus. Systemic spread was directly correlated to the silencing suppressor activity but was independent of the specific nucleolar localization. Interestingly, the RSS activity of p14 was found more active in root than in leaves, which makes sense as BNYVV is a soil-transmissible virus (Andika et al., 2012).

### RNA motifs

Formation of viral RNP complexes and their transport in SE is likely to require the presence of RNA motifs recognized by viral or cellular proteins. RNA sequences critical for systemic infection were first identified for viroids, these unconventional pathogens which do not encode proteins and are transported over long-distances in the form of RNP complexes (Ding, 2009). Specific RNA loops found on the *Potato spindle tuber viroid* (PSTVd) sequence resemble protein-binding sites on rRNAs (Zhong et al., 2007, 2008; Ding, 2009). These structures could potentially be the target site for phloem proteins like the phloem lectin PP2 which was shown to bind viroid RNA *in vitro* and *in vivo* (Gómez and Pallás, 2001, 2004; Owens et al., 2001).

Identification of RNA motifs required for systemic transport is not restricted to viroids and has recently been shown for benyviruses. Although BNYVV RNA-3 is not required for cell-to-cell movement, it is essential for virus vascular movement in *B. macrocarpa*, but not in *S. oleracea* (Tamada and Abe, 1989; Lauber et al., 1998). Using RNA-3 mutants, Lauber et al. (1998) showed that the sequence essential for movement is located in an internal “core” domain of RNA-3 and does not require protein expression. RNA-3, and not the encoded proteins, is therefore described as a host-specific long-distance factor for BNYVV. Interestingly, BNYVV RNA-3 can be successfully replaced by *Beet soil-borne mosaic benyvirus* (BSBMV) RNA-3 for systemic spread in *B. macrocarpa* (Ratti et al., 2009). A fully conserved 22 nucleotides sequence in BNYVV and BSBMV RNA-3 sequences was designated as the “coremin” sequence. This sequence is also present in BNYVV RNA-5, BSBMV RNA-4, as well as in other viral RNA species belonging to the genus *Cucumovirus* (Ratti et al., 2009). It could therefore represent a viral determinant involved in long-distance movement of different viruses. Site directed mutagenesis of the coremin sequence confirmed the role of this sequence in BNYVV systemic spread in *B. macrocarpa* (Peltier et al., 2012). An even more complex view of benyvirus long-distance trafficking can be underlined as BNYVV RSS p14, was found to take part in this function (Chiba et al., 2013; and previous section on RSS). Additional experiments are required to decipher the molecular mechanism by which the coremin sequence affects benyviruses vascular transport and to identify plant and/or viral partners of this RNA sequence.

## Host Determinants Promoting or Restricting Virus Long-Distance Movement

In addition to viral components, host factors can be recruited to assist virus phloem transport. Cellular proteins are potentially involved in the formation of viral complexes and can foster an efficient delivery of such complexes to and through PD. They may also act as stabilizing factors or as protective agents against plant defense mechanisms. Such plant factors were mostly identified by different screens, either genetic using various *A. thaliana* mutants or biochemical using host cDNA libraries in yeast two-hybrid experiments. Host proteins interacting with viral movement determinants and whose implication in virus vascular trafficking has been demonstrated are listed in Table A2 in Appendix. Most of these cellular proteins are usually host and virus-specific, suggesting that more than a unique molecular process governs virus long-distance transport. This implies also that many more factors remain to be discovered, which will certainly help to unravel the mechanisms by which the cellular components assist viral systemic movement.

In contrast to these factors facilitating virus transport, other plant proteins function to restrict virus long-distance movement leading to virus resistance. Information on these specific cellular determinants is still extremely sparse.

### Host determinants that promote virus systemic movement

#### Host factors involved in phloem transport of tobamoviruses

A screen of EMS *A. thaliana* treated plants identified a mutant named *vsm1* (virus systemic movement) in which entry of *Turnip vein clearing virus* (TVCV) into vascular tissue is inhibited (Lartey et al., 1997, 1998). The effect of *vsm1* on virus systemic spread seems to be specific to tobamoviruses because transport of TCV, a carmovirus, is not affected by the *vsm1* mutation, whereas long-distance movement of the tomato strain of *Tobacco mosaic virus* (TMV), another tobamovirus, is restrained in the *A. thaliana* mutant (Lartey et al., 1998). Genetic analysis showed that *vsm1* is a recessive mutation at a single locus that has not been mapped yet. As the systemic movement was likely blocked at the level of entering the vascular tissue in the mutant plant, it was hypothesized that VSM1 could assist virus loading into SE (Lartey et al., 1998).

Another recessive resistance gene to TMV-U1 strain was identified in *A. thaliana* Col-0 and named *DSTM1* for Delayed Systemic Tobamovirus Movement 1 (Pereda et al., 2000). Strikingly, virus particles observed in the vascular tissue of this accession displayed a different morphology (curved virions) than those observed in mesophyll cells of Col-0 or in susceptible accessions (rigid rods) (Serrano et al., 2008). This suggests that *DSTM1* may encode a phloem host factor required for correct virion assembly, virus stability or virus transport in the SE.

In addition to *VSM1* and *DSTM1* genes that have not yet been precisely mapped, two known cellular proteins have been shown to participate in long-distance transport of tobamoviruses. Pectin methylesterase (PME), a cell wall protein of tobacco required for cell-to-cell movement of TMV, may also assist virus long-distance transport (Chen et al., 2000; Chen and Citovsky, 2003). Specific inhibition of PME expression in tobacco tissues led to a significant delay of TMV systemic infection. Immunofluorescence confocal microscopy observations of inoculated *PME*-silenced plants showed that the virus is loaded into the host vasculature, but is inefficiently unloaded from the phloem into non-inoculated leaves. These results infer that virion entry into and exit from vasculature are controlled by two different mechanisms, and PME could act at the level of virus egress from SE. Whether an interaction between PME and TMV MP is required for virus phloem unloading has not been addressed (Chen and Citovsky, 2003).

The other protein affecting long-distance transport of tobamoviruses is the IP-L protein of 16.8 kDa, an “elicitor responsive protein,” also related to senescence. IP-L was identified by screening a tobacco cDNA library using a yeast two-hybrid assay with the CP of *Tomato mosaic virus* (ToMV) as a bait. Repression of IP-L expression by virus-induced gene silencing (VIGS) led to a delay in virus accumulation in non-inoculated leaves. This suggests that a high expression level of IP-L is important for efficient ToMV systemic infection (Li et al., 2005), a hypothesis sustained by the increased IP-L mRNA accumulation observed in ToMV infected *N. tabacum* (Li et al., 2005). The mechanism by which IP-L affects viral systemic movement is still unknown.

#### Host factors involved in phloem transport of potyviruses

Using a yeast two-hybrid system screen, a cellular factor interacting with the VPg proteins of different potyviruses was identified from pea and named PVIP for *Potyvirus* VPg-interacting protein (Dunoyer et al., 2004). The PVIP orthologs in *N. benthamiana* and *A. thaliana* exhibit the same ability to bind potyvirus VPg proteins. The VPg determinants involved in the binding are located in the N-terminal part of the protein. The PVIP/VPg interaction was shown to be important for virus movement, as mutations in the VPg sequence preventing its interaction with PVIPs strongly reduced TuMV local and systemic movement (Dunoyer et al., 2004). However, it is not clearly determined whether the effect on long-distance movement is a direct consequence or an indirect effect due to slower cell-to-cell movement. Reduction of PVIP expression in transgenic RNAi lines showed that PVIP is not required for virus replication, but functions as an ancillary factor for potyvirus movement. PVIP is part of a small gene family of *A. thaliana* whose proteins contain a plant homeodomain (PHD) with the capacity to regulate gene expression through histone modifications (reviewed in Cosgrove, 2006). The *Arabidopsis* PVIP2 and PVIP1 correspond to OBERON1 (OBE1) and OBERON2 (OBE2) respectively, which were described as having redundant functions in the establishment and/or maintenance of the shoot and root apical meristems (Saiga et al., 2008; Thomas et al., 2009). They also act as central regulators in auxin-mediated control of development (Thomas et al., 2009). The nuclear localization of both VPg (Restrepo et al., 1990; Rajamäki and Valkonen, 2009) and PVIP factors (Saiga et al., 2008) raises the possibility that PVIP/VPg interaction may modulate expression of host genes involved in virus movement.

A resistance screen of several *A. thaliana* accessions identified a recessive resistance gene, referred to as *SHA3*, which strictly restricts PPV long-distance movement. By genetic linkage and genome-wide association analyses, the gene was positioned at the bottom of chromosome 3 in a cluster of 13 genes encoding *RTM3* (a resistance gene involved in the restriction of potyvirus long-distance movement; see below) and *RTM3*-likes genes (Pagny et al., 2012). However, the two genes *SHA3* and *RTM3*, both involved in potyvirus long-distance transport, were shown to be distinct genes. The cloning of *SHA3* will be an important breakthrough, as it will represent the first identified susceptibility factor directly involved in potyvirus systemic movement.

#### Role of the nucleolar fibrillarin in viral long-distance movement

There is growing evidence that fibrillarin, a major nucleolar protein essential for RNA processing, functions in long-distance transport of RNA viruses. This implies a nucleolar phase in the virus life cycle, which is the case for umbraviruses (see above; Taliansky et al., 2010). *A. thaliana* fibrillarin expression knockdown by RNA silencing did not affect umbravirus replication, nor virus cell-to-cell movement, but inhibited virus long-distance transport (Kim et al., 2007b). Fibrillarin interacts directly with the GRV long-distance movement factor (ORF3 protein) and this interaction induces a redistribution of the fibrillarin/ORF3 protein complexes in the cytoplasm. Such complexes associate with viral RNA to form RNP particles which are then transported from cell-to-cell, and ultimately loaded into SE for virus systemic movement (Kim et al., 2007a). Implication of fibrillarin in virus long-distance trafficking is likely not limited to umbraviruses because PLRV (*Polerovirus*) is unable to move systemically in fibrillarin-silenced plants, while viral accumulation in inoculated leaves remains unaffected (Kim et al., 2007b). Viruses from the *Virgaviridae* family, like PMTV and PSLV, represent other examples for which the MP TGB1 localizes to the nucleolus and interacts with fibrillarin (see above; Wright et al., 2010; Semashko et al., 2012a). Whether fibrillarin is involved in long-distance movement of these viruses has not been established yet.

#### Role of Tcoi1 and PP1 in Cucumber mosaic virus movement

A yeast two-hybrid screen of a *N. tabacum* cDNA library with the CMV-1a protein, a replication essential protein, led to the identification of the *Tcoi1* gene product (Kim et al., 2008). Tcoi1 protein contains a methyltransferase domain that interacts with the CMV-1a protein leading to methylation of the viral protein (Kim et al., 2008). When over-expressed in transgenic plants, Tcoi1 protein increased CMV RNA accumulation in non-inoculated leaves. The opposite effect was observed in transgenic plants where *Tcoi* expression was reduced. Conversely, CMV infectivity was not affected by Tcoi1 in inoculated leaves, supporting that Tcoi1 influences CMV long-distance movement (Kim et al., 2008). Overall, these data suggest that protein methylation is crucial for CMV-1a function, thereby facilitating viral replication and/or systemic movement.

P48 is another protein, identified in *C. sativus* phloem exudate, potentially involved in CMV long-distance transport and showing virus-binding capacity (Requena et al., 2006). P48 is homologous to *Cucurbita maxima* PP1, a 96 kDa protein synthesized in CC, found in P-protein filaments together with PP2, and which can be translocated with the phloem stream (Requena et al., 2006). Interaction between p48 and CMV viral particles is partially responsible for the increased resistance of virions to RNase A when they are mixed with phloem exudate (Requena et al., 2006). Based on these results, it is conceivable that CMV virions-p48 complexes could be important for CMV particle stability, virion release into SE or virion transport in the sap.

### Host determinants which restrict virus long-distance movement

Virus resistance can be achieved by blocking virus long-distance movement. A few examples of such resistance have been genetically characterized for several viruses (Caranta et al., 1997, 2002; Mahajan et al., 1998; Revers et al., 2003; Decroocq et al., 2006; Maule et al., 2007), but only few genes were identified by cloning.

#### cdiGRP and callose deposition

Experiments performed by Ueki and Citovsky (2002) showed that TMV and TVCV (*Tobamovirus*) systemic movement was reduced in tobacco plants treated specifically with low but not with high concentrations of the heavy metal cadmium. Using cDNA library subtraction experiments, a glycine-rich protein (GRP), which expression is specifically induced by low cadmium level, was identified and named cadmium-ion-induced GRP protein (cdiGRP). This vascular protein is localized in the cell wall of SE and CC. It contains an amino-terminal secretion signal, an internal glycine-rich domain and a carboxy-proximal cysteine-rich domain which could be responsible for protein cell wall targeting. Antisense expression of the cdiGRP cDNA in tobacco plants reduced cdiGRP mRNA accumulation in cadmium-treated plants, and allowed systemic movement of TVCV. Conversely, over-expression of cdiGRP reduces TVCV systemic movement by preventing the exit of virions from vascular bundles, which reinforces the role of cdiGRP in restricting virus long-distance trafficking. The blocking capacity of cdiGRP may be explained by callose deposition in the cell wall of phloem cells observed after cadmium treatment, or after constitutive expression of cdiGRP.

#### RTM genes

A genetic screen of EMS-mutagenized *A. thaliana* Col-0 populations identified several mutants allowing long-distance movement of TEV in an ecotype that normally restricts the virus to inoculated leaves. The identified *RTM* (for Restricted TEV Movement) resistance genes are dominant and effective against several potyviruses (Mahajan et al., 1998; Revers et al., 2003; Decroocq et al., 2006). In this resistance process, viral replication and cell-to-cell movement in inoculated leaves are not affected, HR and systemic acquired resistance (SAR) are not triggered and salicylic acid is not involved (Mahajan et al., 1998). Genetic characterization of natural *A. thaliana* accessions and *A. thaliana* mutants showed that at least five dominant genes, named *RTM1*, *RTM2*, *RTM3*, *RTM4*, and *RTM5*, are involved in this resistance (Mahajan et al., 1998; Whitham et al., 1999; Cosson et al., 2012). A single mutation in one of the RTM genes is sufficient to abolish the resistance phenotype (Whitham et al., 1999). RTM1 encodes a protein belonging to the jacalin family (Chisholm et al., 2000). RTM2 expresses a protein with similarities to small heat shock proteins and contains a transmembrane domain (Whitham et al., 2000). RTM3 belongs to a meprin and TRAF homology (MATH) domain protein family, and possesses a coiled-coil domain at its C-terminal end. In addition, it was shown that RTM3 interacts with RTM1 (Cosson et al., 2010). RTM4 and RTM5 have only been genetically characterized (Cosson et al., 2012). RTM1 and RTM2 are specifically expressed in phloem-associated tissues and the corresponding proteins localize to SE (Chisholm et al., 2001). Despite the fact that the CP of potyviruses is the viral determinant overcoming the RTM resistance (Decroocq et al., 2009), none of the RTM proteins has been found to interact with CP (Cosson et al., 2010). However, interaction between CP, or whole virions, with RTM proteins mediated by additional cellular or viral proteins is still conceivable. Indeed, self- and cross-interactions of RTM1 and RTM3 were observed which suggest that these proteins may be part of a larger protein complex (Cosson et al., 2010). Different hypothesis can be proposed regarding the RTM resistance mechanism: (i) virus particles, in the process of being loaded into SE, could be sequestered by the RTM complex; (ii) the RTM complex could reduce virus accessibility to cellular factor(s) or structure(s) required for potyvirus long-distance movement; (iii) RTM complex could activate a movement-restricting response of the plant following virus infection.

#### Proteolysis

A study on PVX long-distance movement suggested an unexpected role of protein degradation in viral phloem exit. PVX TGB1 and CP were fused to GFP and expressed in transgenic *N. benthamiana* under the control of a CC-specific promoter (Mekuria et al., 2008). Whereas the fusion proteins were largely confined to the vasculature in petioles and leaves, indicating their inability to exit the phloem, they spread into mesophyll cells in plants treated with proteasome inhibitors. A similar effect was observed in plants infected with PVX. These data raise the intriguing hypothesis that proteolysis could play a role in restricting viral proteins in the phloem, and that PVX has the ability to protect its own proteins from proteasomal degradation (Mekuria et al., 2008). Further molecular and genetic studies are required to decipher the underlying mechanism. These data point out that the proteasome degradation process may be active in the phloem which is in accordance with the detection of many proteasome components in the SE (Lin et al., 2009; Dinant and Lemoine, 2010). Another study by Jin et al. (2006) showed that down-regulation of the 26S proteasome subunit RPN9 alters vascular development and leads to inhibition of viral systemic infection. However, the effect on virus long-distance movement could also originate from pleiotropic effects due to alteration of the plant vasculature.

#### SA-mediated defense response

In addition to its essential role in the development of the hypersensitive response (HR) and the SAR (Vlot et al., 2009), salicylic acid (SA) may restrict long-distance movement of plant viruses as exemplified in several studies.

In tobacco and in *A. thaliana* plants treated with SA, CMV systemic movement is delayed whereas virus replication and cell-to-cell movement are unaffected in inoculated leaves. This SA-induced inhibition of CMV systemic movement involves the mitochondrial signaling pathway (Naylor et al., 1998; Mayers et al., 2005). Ji and Ding (2001) showed that systemic movement of a CMV mutant that does not express the RSS 2b, was completely blocked by SA treatment in young *N. glutinosa* seedlings, while the wild-type CMV spread was unaffected. This assay evidenced that the CMV 2b protein antagonized the SA-based host defense mechanism. However, the RSS activity of the CMV 2b protein (see above section) may overlap the effect on SA-resistance. Lewsey and Carr (2009) showed that in *A. thaliana* DCLs 2, 3, and 4 are dispensable for SA-induced resistance to CMV which means that the RNA silencing pathway controlled by these three DCLs is not involved in SA-induced resistance.

Another evidence showing that SA is involved in virus long-distance transport comes from PPV inoculation experiments on *N. tabacum* plants. Although PPV replicates and moves from cell-to-cell in the inoculated leaves, it cannot reach non-inoculated leaves in this host (Sáenz et al., 2002; Alamillo et al., 2006). However, PPV was able to move systematically in transgenic tobacco plants expressing either HC-Pro of TEV or the *NahG* gene encoding the bacterial salicylate hydroxylase, a SA-degrading enzyme (Alamillo et al., 2006). Interestingly, double transgenic plants expressing both TEV HC-Pro and the *NahG* gene showed increased spread of PPV, suggesting that RNA silencing and SA-mediated defense have additive effects on PPV infection.

Finally, inhibition of CaMV long-distance movement was also observed in *cpr1* and *cpr5 A. thaliana* mutants possessing a constitutive over-expression of SA due to the absence of negative regulators of the SA metabolic pathway (Love et al., 2007). Transgenic *A. thaliana* CaMV-encoded RSS P6 protein showed repression of SA-responsive genes, inferring that P6 may inhibit SA-mediated effect (Love et al., 2012). However, SA-deficient *A. thaliana* mutants did not exhibit enhanced susceptibility to CaMV. A plausible mechanism for the enhanced resistance of *cpr* mutants to CaMV could be that, as already suggested in some examples (Xie et al., 2001; Alamillo et al., 2006), SA-dependent defense responses may act synergistically with RNA silencing. These controversial data may arise from experimental conditions settings or from the different mechanisms of action of the RSS. Further investigations are required to shed light on these intricate pathways.

## Impact of Virus Transport in the Phloem on Virus Titer and Diversity

As previously mentioned, long-distance movement of plant viruses is composed of different steps comprising virus loading (entry) into the phloem tissue, virus movement inside SE, and virus unloading (exit) into cells of the sink tissue. Each of these steps represents a potential barrier for virus trafficking and several examples of viruses blocked at one stage or another were described in this review. Whether the crossing of such cell interfaces induces bottlenecks in a virus population constitutes a new challenge for the future because data on this issue are still sparse. The first quantitative analysis of a virus population in the vasculature has recently being conducted on CaMV using measures of virus titer in aphids as a reflection of virus load in the sap (Gutiérrez et al., 2012a,b). Whereas CaMV overall concentration in the different leaves was relatively constant, the number of genome copies circulating in the sap varied depending of the leaf stage: the number of viral genome increased progressively as the infection progresses and after reaching a maximum, it decreases in the youngest leaves late in infection. The virus titer within the plant vasculature correlates with the multiplicity of cellular infection (the number of viral genomes entering and replicating within a cell) among leaf levels (Gutiérrez et al., 2010, 2012a). In this specific case, the bottleneck is driven by the virus load in the sap. Several hypotheses were raised by the authors to explain this drop in virus load late in infection like an arrest of virion export from infected leaves, an increased virus degradation rate within the sap or a massive storage of virions in unknown plant compartments.

However, evolution of a viral population in infected plants does not seem to always fit the CaMV model. For instance, the viral population, or the genetic bottlenecks, may not be related to the amount of viruses circulating in the sap, but may be rather driven by physical host barriers like the structure of the minor veins or the characteristics of the PD. This situation is exemplified by CMV for which a constant loss of genetic diversity was observed all along the infection (Li and Roossinck, 2004; Ali and Roossinck, 2010). Therefore, in this particular case, virus long-distance transport plays a significant role in reducing virus population variation. Interestingly, these studies on CMV also highlighted the high impact of the host on the genetic bottlenecks, which may explain the virus population diversity in different hosts.

From these data, it has been suggested that the size of the virus population invading the sink organs from vasculature depends either on the concentration of virus in sap or on barriers imposed by the host (Gutiérrez et al., 2012b). However, it is very likely that a range of intermediate situations exists between these two opposite scenarios and more efforts are now required to better understand the viral population dynamics in vasculature for a wide and diverse panel of virus species.

## Concluding Remarks

In the last two decades many viral determinants involved in systemic invasion of plants have been identified or better characterized. It is now well established that beside the CP, many non-structural viral proteins (MP, TGB1, VPg, RSS, …) and even structural motifs on viral RNA are implicated in virus long-distance transport. It becomes also clearer that in addition to the predominant virions, RNP complexes constitute an important form of long-distance spread and that several forms of viral devices can even co-exist, in the same host. Understanding whether there is a specialization of these different forms, either in the time course of infection or for the crossing of the various cell borders or even in some specific environments (host species, developmental stage, or stress of the plant) will be a serious challenge. Plant physiology and virology are associated disciplines that should provide reciprocal feedbacks for the understanding of transport processes in phloem. In particular, a better knowledge of the structure and function of the various cell types composing the vascular tissues and the specialized PD at each cell interface would be greatly beneficial for virology. In addition, a deeper characterization of plant defense responses (RNAi, SA-mediated resistance) induced during virus systemic movement is necessary to decipher their molecular mechanisms and their connexions with the viral life cycle. Finally, although many RSS with apparently very diverse modes of action were identified for most viruses, their precise involvement in virus systemic spread remains an essential issue. Among others, the question of how and where viral RSSs interfere with the movement of the extremely abundant siRNA is puzzling.

An emerging field of research that appeared very recently concerns the size of virus population moving in the SE and able to invade the distant organs from the vasculature. Regulation of the virus population through the phloem involves very tightly regulated barriers that are essential check-points for plant development (Dinant and Lemoine, 2010). By restricting the flow of photoassimilates, the plant may regulate at the same time dispersal of pathogens throughout the plant. From the virus point of view, regulating the population dynamics in the vasculature has profound consequences on virus transmission by phloem feeding insects but also on virus evolution. However, investigations on additional virus models than the one studied so far will be necessary to get a broader view on the influence of viral long-distance movement on the epidemiology of virus diseases.

Finally from an agronomical point of view, identification of plant proteins required for viral systemic movement can potentially generate new sources of virus resistance in crops. Selecting from natural populations or by genetic engineering plants deficient for cellular proteins required in viral cycle is an efficient strategy to develop recessive resistance genes against viruses (Maule et al., 2007; Gómez et al., 2009; Truniger and Aranda, 2009; Wang and Krishnaswamy, 2012). In particular, the advent of new technologies such as Targeting-Induced Local Lesions IN Genome (TILLING), EcoTILLING, high-resolution melting (HRM), KeyPoint and next-generation sequencing, may boost the identification of target gene mutants from artificially induced mutant libraries or natural populations, especially in agronomically important crops (Nieto et al., 2007; Hofinger et al., 2009; Rigola et al., 2009; Ibiza et al., 2010; Piron et al., 2010). Genetic resistance can also be generated by silencing or overexpressing the candidate genes depending on the beneficial or the detrimental role of the cellular protein involved in virus long-distance movement (Wang and Krishnaswamy, 2012; Wang et al., 2013). However, these strategies may be of limited use as they may also strongly affect plant macromolecule transport and consequently plant development. Nevertheless, the current lack of efficient methods to restrict or eradicate plant viruses should foster the exploration of these new strategies.

## Conflict of Interest Statement

The authors declare that the research was conducted in the absence of any commercial or financial relationships that could be construed as a potential conflict of interest.

## Appendix

**Table A1:** **Viral proteins and transport complexes involved in virus long-distance movement**.

Genus	Capsid structure	Genome	Viral species	Viral factors required for long-distance movement	Viral form used for long-distance movement	Hosts	Reference
**ALPHAFLEXIVIRIDAE**							
*Potexvirus*	Helical capsid	ssRNA + monopartite	*White clover mosaic virus* (WCMV)	CP, TGB1	*RNP*	*N. benthamiana*	Lough et al. (2001)
			*Potato virus X* (PVX)	CP	Virions	*N. benthamiana*	Betti et al. (2011), Cruz et al. (1998)
**BROMOVIRIDAE**							
*Alfamovirus*	Icosahedral capsid	ssRNA + multipartite	*Alfalfa mosaic virus* (AMV)	CP	Virions	*N. benthamiana, N. tabacum, S. oleracea*	Herranz et al. (2012), Spitsin et al. (1999), Tenllado and Bol (2000)
*Cucumovirus*	Icosahedral capsid	ssRNA + multipartite	*Tomato aspermy virus* (TAV)	CP	*Virions*	*C. sativus, N. tabacum, N. clevelandii*	Llamas et al. (2006), Salánki et al. (2011), Taliansky and García-Arenal (1995)
			*Cucumber mosaic virus* (CMV)	CP, MP	Virions	*C. sativus, N. tabacum*, Squash, *N. clevelandii*	Llamas et al. (2006), Requena et al. (2006), Salánki et al. (2011), Taliansky and García-Arenal (1995), Thompson et al. (2006); Ueki and Citovsky (2007); Waigmann et al. (2004)
				2b		*C. sativus*	Brigneti et al. (1998), Ding et al. (1995), Guo and Ding (2002)
*Bromovirus*	Icosahedral capsid	ssRNA + multipartite	*Brome mosaic virus* (BMV)	CP, MP	*RNP*, Virions	*C. hybridum, H. vulgare, N. benthamiana*	Flasinski et al. (1997), Gopinath and Kao (2007), Kao et al. (2011), Okinaka et al. (2001), Sacher and Ahlquist (1989), Takeda et al. (2004)
			*Cowpea chlorotic mottle virus* (CCMV)	CP	?	*N. benthamiana, H. vulgare, V. unguiculata*	Allison et al. (1990), Schneider et al. (1997)
**BUNYAVIRIDAE**							
*Tospovirus*	Icosahedral enveloped capsid	Ambisense ssRNA multipartite	*Tomato spotted wilt virus* (TSWV)	N (CP), NSm	*RNP*	*N. benthamiana, N. tabacum*	Lewandowski and Adkins (2005) Li et al. (2009), Zhang et al. (2011)
**CLOSTEROVIRIDAE**							
*Closterovirus*	Helical capsid	ssRNA, monopartite	*Beet yellows virus* (BYV)	CP, p20, p21, L-Pro	Virions	*N. benthamiana, A. thaliana*	Prokhnevsky et al. (2002), Peng et al. (2003), Peremyslov et al. (2004), Chapman et al. (2004)
			*Citrus tristeza virus* (CTV)	p20, p33, p18, p13	?	*Citrus* species	Tatineni et al. (2008), Folimonova et al. (2008), Tatineni et al. (2011b)
**GEMINIVIRIDAE**							
Monopartite and Bipartite *Geminiviridae*				MP			Reviewed in Waigmann et al. (2004), Ueki and Citovsky (2007)
*Mastrevirus*	Icosahedral capsid	ssDNA monopartite	*Maize streak virus* (MSV)	CP	Virions	*Zea mays*	Boulton et al. (1989), Liu et al. (2001)
*Curtovirus*	Icosahedral capsid	ssDNA monopartite	*Beet mild curly top virus* (BMCTV)	CP	*Virions*	*N. benthamiana*	Soto et al. (2005)
*Begomovirus*	Icosahedral capsid	ssDNA monopartite or bipartite	*Tomato golden mosaic virus* (TGMV)	ΔCP, ?	RNP*	*N. benthamiana*	Jeffrey et al. (1996), Pooma et al. (1996)
				CP	*Virions*	*N. benthamiana, N. tabacum, D. stramonium*	
			*Bean golden mosaic virus* (BGMV)	ΔCP, ?	RNP*	*Phaseolus vulgaris*	Pooma et al. (1996)
				CP	*Virions*	*Phaseolus vulgaris N. benthamiana*	
			*Tomato yellow leaf curl virus* (TYLCV)	CP	Virions	*N. benthamiana*	Noris et al. (1998), Wartig et al. (1997)
			*Tomato leaf curl virus* (ToLCV) – India	ΔCP, ? CP	RNP* *Virions*	*N. benthamiana, S. lycopersicum*	Padidam et al. (1995, 1996)
**LUTEOVIRIDAE**							
*Polerovirus*	Icosahedral capsid	ssRNA + monopartite	*Turnip yellows virus* (TuYV)	CP, RT, RT*, P4	*Virions*	*C. quinoa, N. clevelandii*	Brault et al. (2000, 2003), Bruyère et al. (1997), Ziegler-Graff et al. (1996)
			*Potato leafroll virus* (PLRV)	CP, RT, RT*, P4	*Virions*	*N. benthamiana, N. clevelandii, P. floridana, S. tuberosum*	Kaplan et al. (2007), Lee et al. (2002, 2005), Peter et al. (2008)
**POTYVIRIDAE**							
*Potyvirus*	Helical capsid	ssRNA + monopartite	*Tobacco etch virus* (TEV)	CP, VPg, HC-Pro	?	*N. tabacum*	Dolja et al. (1994), Cronin et al. (1995), López-Moya and Pirone (1998), Schaad et al. (1997), Kasschau and Carrington (2001)
			*Potato virus A* (PVA)	CP, VPg, 6K2	?	*N. physaloides, S. commersonii*	Hämäläinen et al. (2000), Ivanov et al. (2003), Rajamäki and Valkonen (1999, 2002)
**TOMBUSVIRIDAE**							
*Dianthovirus*	Icosahedral capsid	ssRNA + multipartite	*Red clover necrotic mosaic virus* (RCNMV)	MP			Reviewed in Waigmann et al. (2004), Ueki and Citovsky (2007)
				CP	Virions	*N. benthamiana, N. clevelandii*	Vaewhongs and Lommel (1995), Xiong et al. (1993)
				ΔCP, ?	RNP	*N. benthamiana* (15°C)	
				ΔCP, ?	RNP*	*N. benthamiana*	Park et al. (2012)
			*Carnation ringspot virus* (CRSV)	CP	Virions	*N. benthamiana*	Sit et al. (2001)
*Tombusvirus*	Icosahedral capsid	ssRNA + monopartite	*Tomato bushy stunt virus* (TBSV)	ΔCP, ?	RNP*	*N. benthamiana, N. clevelandii*	Desvoyes and Scholthof (2002), Qu and Morris (2002), Scholthof et al. (1993)
				CP	Virions		
				P19		*S. oleracea, Capsicum*	Dunoyer et al. (2010), Scholthof et al. (1995), Voinnet et al. (1999)
			*Cymbidium ringspot virus (CymRSV)*	ΔCP, ?	RNP	*N. benthamiana*	Dalmay et al. (1992)
				CP	Virions	*N. clevelandii*	
				P19		*N. benthamiana*	Havelda et al. (2003)
*Carmovirus*	Icosahedral capsid	ssRNA + monopartite	*Turnip crinkle virus* (TCV)	CP	Virions	*B. campestris, N. benthamiana, A. thaliana*	Heaton et al. (1991), Cohen et al. (2000), Qu et al. (2003), Choi et al. (2004), Deleris et al. (2006), Cao et al. (2010)
*Necrovirus*	Icosahedral capsid	ssRNA + monopartite	*Olive latent virus* (OLV-1)	CP	Virions	*N. benthamiana*	Pantaleo et al. (1999, 2006)
**VIRGAVIRIDAE**							
*Tobamovirus*	Helical capsid	ssRNA + monopartite	*Tobacco mosaic virus* (TMV)	CP	Virions	*N. sylvestris, N. tabacum*	Holt and Beachy (1991), Knorr and Dawson (1988), Osbourn et al. (1990), Saito et al. (1990)
*Hordeivirus*	Helical capsid	ssRNA + multipartite	*Barley stripe mosaic virus* (BSMV)	TGB1	RNP	*H. vulgare, N. benthamiana, C. quinoa*	Jackson et al. (2009), Lim et al. (2008), Makarov et al. (2009), Petty et al. (1990), Solovyev et al. (2012), Verchot-Lubicz et al. (2010)
*Pomovirus*	Helical capsid	ssRNA + multipartite	*Potato mop-top virus* (PMTV)	ΔCP, TGB1	RNP (RNA1 and 2 only)	*N. benthamiana, N. clevelandii*	McGeachy and Barker (2000), Savenkov (2003), Torrance et al. (2009, 2011), Wright et al. (2010)
				CP, CP-RT, TGB1	Virions	
*Tobravirus*	Helical capsid	ssRNA + multipartite	*Tobacco rattle virus* (TRV)	ΔCP, ? (NM isolates)	RNP*	*N. benthamiana, N. clevelandii*	Macfarlane (2010), Swanson et al. (2002), Torrance et al. (2011), Wright et al. (2010), Ziegler-Graff et al. (1991)
				CP (M isolates)	Virions	
**UNASSIGNED FAMILY**							
*Umbravirus*	Icosahedral capsid	ssRNA + monopartite	*Groundnut rosette virus* (GRV)	P3	RNP	*N. benthamiana, C. quinoa*	Canetta et al. (2008), Kim et al. (2007a,b), Ryabov et al. (2001), Taliansky et al. (2003)
			*Pea enation mosaic virus-2* (PEMV-2)	P3	RNP	*N. benthamiana*	Kim et al. (2007a,b), Ryabov et al. (2001)
*Sobemovirus*	Icosahedral capsid	ssRNA + monopartite	*Southern bean mosaic virus* (SBMV)	CP	Virions	Bean, *V. unguiculata*	Fuentes and Hamilton (1993)
*Benyvirus*	Helical capsid	ssRNA + multipartite	*Beet necrotic yellow vein virus* (BNYVV)	CP, P14	Virions	*C. quinoa, T. expansa*	Chiba et al. (2013), Lauber et al. (1998), Peltier et al. (2012), Quillet et al. (1989), Ratti et al. (2009)
				Coremin sequence		*B. macrocarpa*	
*Betaflexiviridae, Caulimoviridae, Nanoviridae, Rhabdoviridae, Secoviridae, Tymoviridae*							
No reference on long-distance movement							

*Viral proteins, described in the review and involved in virus long-distance movement, are listed in the table (CP: capsid protein; N: nucleocapsid protein; MP: movement protein; TGB: triple gene block protein; VPg: viral protein genome-linked; RT: readthrough protein; RT*: truncated form of RT; HC-Pro: helper component-proteinase). The viral complex transported over long-distances [virions or ribonucleoprotein (RNP) complexes], is also indicated when clearly identified. For some viruses, uncertainties still remain concerning the nature of the viral form moving systemically. In these cases, the most likely form of transport is indicated in *italic*s. In some cases, in addition to virions, an alternative form of virus transport can be observed when the CP is deleted or mutated (ΔCP) but the systemic movement of these RNP complexes (RNP*) is usually less efficient than the one involving virions*.

**Table A2:** **Host factors known or suspected to be involved in virus long-distance transport**.

Virus species	Host factors involved on viral long-distance movement		Host plants	Reference
	Positive effect	Negative effect	
**BROMOVIRIDAE (CUCUMOVIRUS)**				
*Cucumber mosaic virus* (CMV)	Tcoi1		*N. tabacum, N. benthamiana*	Kim et al. (2008)
	p48 (PP1)		*C. sativus*	Requena et al. (2006)
		Salicylic acid	*A. thaliana*	Naylor et al. (1998), Ji and Ding (2001), Mayers et al. (2005), Lewsey and Carr (2009)
**CAULIMOVIRIDAE (CAULIMOVIRUS)**				
*Cauliflower mosaic virus* (CaMV)	PME		*N. tabacum*	Chen et al. (2000)
		Salicylic acid	*A. thaliana*	Xie et al. (2001), Alamillo et al. (2006), Love et al. (2007, 2012)
**LUTEOVIRIDAE (POLEROVIRUS)**				
*Potato leafroll virus* (PLRV)	Fibrillarin		*A. thaliana*	Kim et al. (2007a,b)
**POTYVIRIDAE (POTYVIRUS)**				
*Pea seed-borne mosaic virus* (PSbMV), *Lettuce mosaic virus* (LMV), *Turnip mosaic virus* (TuMV)	PVIPnb		*N. benthamiana*	Dunoyer et al. (2004), Saiga et al. (2008)
	AtPVIP (OBERON)		*A. thaliana*	
	PVIPp		*P. sativum*	
*Lettuce mosaic virus* (LMV), *Plum pox virus* (PPV), *Tobacco etch virus* (TEV)		RTM genes	*A. thaliana*	Cosson et al. (2012), Decroocq et al. (2006), Mahajan et al. (1998), Revers et al. (2003), Whitham et al. (1999)
*Plum pox virus* (PPV)	SHA3		*A. thaliana*	Pagny et al. (2012)
		Salicylic acid	*N. tabacum*	Alamillo et al. (2006), Sáenz et al. (2002)
**VIRGAVIRIDAE (TOBAMOVIRUS, POMOVIRUS)**				
*Turnip vein clearing virus* (TVCV)	VSM1		*A. thaliana*	Lartey et al. (1997, 1998)
	PME		*N. tabacum*	Chen et al. (2000)
		cdiGRP	*N. tabacum*	Ueki and Citovsky (2002)
*Tobacco mosaic virus* (TMV)	DSTM1		*A. thaliana*	Pereda et al. (2000), Serrano et al. (2008)
	VSM1		*A. thaliana*	Lartey et al. (1998)
	PME		*N. tabacum*	Chen and Citovsky (2003), Chen et al. (2000)
		cdiGRP	*N. tabacum*	Ueki and Citovsky (2002)
*Tomato mosaic virus* (ToMV)	IP-L		*N. tabacum*	Li et al. (2005)
*Potato mop-top virus* (PMTV), *Poa semilatent virus* (PSLV)	Fibrillarin		*A. thaliana*	Semashko et al. (2012b), Wright et al. (2010)
**UNASSIGNED FAMILY (UMBRAVIRUS, TENUIVIRUS)**				
*Groundnut rosette virus* (GRV)	Fibrillarin		*A. thaliana*	Kim et al. (2007a,b)

*Host factors, described in the review for their beneficial or antagonist action on virus long-distance movement, are listed in the table*.
